# Power–Load Relationship of Bench Press, Ballistic Bench Press, and Prone Bench Pull in International Medal-Winning Canoeists and Kayakers

**DOI:** 10.3390/sports13060191

**Published:** 2025-06-19

**Authors:** Oscar Crisafulli, Matteo Fortunati, Tiziano Gemelli, Massimiliano Febbi, Patrik Drid, Stefano Ramat, Giuseppe D’Antona

**Affiliations:** 1 Centro di Ricerca Interdipartimentale Attività Motorie e Sportive (CRIAMS)-Sport Medicine Centre Voghera, University of Pavia, 27058 Voghera, Italy; oscar.crisafulli@unipv.it (O.C.); matteo.fortunati01@gmail.com (M.F.); tiziano.gemelli@unipv.it (T.G.); 2 Faculty of Sport and Physical Education, University of Novi Sad, 21000 Novi Sad, Serbia; patrikdrid@gmail.com; 3 Department of Industrial Engineering, University of Roma “Tor Vergata”, 00133 Roma, Italy; massimilianofebbi@gmail.com; 4 Laboratory for Rehabilitation, Medicine and Sport (LARM), 00133 Roma, Italy; 5 Department of Electrical, Computer and Biomedical Engineering, University of Pavia, 27100 Pavia, Italy; stefano.ramat@unipv.it; 6 Department of Public Health, Experimental and Forensic Medicine, University of Pavia, 27100 Pavia, Italy

**Keywords:** resistance training, velocity-based training, paddle sport, linear encoder

## Abstract

Paddler athletes use resistance training (RT) to optimize power output (PO) during competitions. Understanding the power–load relationship (P–Lr) is essential for effective RT prescription. Moreover, the push-to-pull ratio (PU/PR)—the one-repetition maximum (1RM) of a pulling exercise divided by the one of a pushing exercise—has been suggested as a metric associated with sprint kayak performance. This study aimed to describe P–Lr in three guided exercises (bench press (BP), ballistic bench press (BBP), and prone bench pull (PBP)), along with PU/PR in international-level canoeing and kayaking athletes. Nine male athletes (21.0 ± 1.5 years) were monitored during two sessions of an incremental testing protocol. Load ranged from 30 to 100 kg in BP, 30 to 95 kg in PBP, and 20 to 60 kg in BBP. Instantaneous displacement was measured using a linear position transducer, and PO was computed for each repetition and exercise. PU/PR was calculated upon PBP and BP. A two-way repeated-measures ANOVA was used to explore differences among exercises and relative load from 20% to 90% 1RM. PBP displayed a higher PO between 40% and 90% 1RM compared to BP and BBP), while no statistical difference was found between BP and BBP at any relative load. Additionally, mean PU/PR resulted 0.96. This study provides preliminary values regarding P–Lr and PU/PR in elite paddlers, which may assist in designing training programs for those targeting major competitions.

## 1. Introduction

Canoe and kayak flat water competitions can vary between an effort of approximately thirty seconds (200 m sprint races) and 1 h 45 min (20 km marathon races). Even if athletes specialize either in short or long races, they must have, at the same time, a high level of maximal strength, power output (PO) and muscular endurance [1,2,3,4]. The PO is necessary for a successful outcome; in fact, a recent investigation found that international medal-winning canoe sprinters have a slightly higher PO than their non-trophy counterparts in resistance training (RT) exercises [3].

Power–load relationship (P–Lr) is a curve that, for a given subject, associates a value of PO (W) to any load (Kg) of a lifting exercise, showing a unimodal, bell-shaped trend [5]. It differs between the type of exercise (i.e., upper or lower-body) [6,7,8,9], gender [10], and age [11]. Measuring P–Lr is essential for trainers to prescribe PO-oriented training to improve a specific part of the competition. For example, knowing that kayak paddlers during a 2 min kayak ergometer test show a maximum PO during an all-out start of 747 ± 151 W and an average 2 min PO of 348 ± 47 W [12], coaches can prescribe a load of a specific resistance training exercise that mimics these PO to further improve them. 

The RT sessions of canoeists and kayakers’ athletes frequently involve bench press (BP), prone bench pull (PBP), and ballistic bench press (BBP). Even if such exercises have been previously used to study P–Lr in other populations of athletes [13,14], to the best of our knowledge, no studies have utilized this paradigm to investigate P–Lr in elite canoeists and kayakers. 

Moreover, in paddle sports, the push-to-pull ratio (PU/PR) obtained by dividing the 1RM of bench pull or weighted chin-up (PU) against the 1RM of bench press (PR) or similar exercises is a valuable information. Indeed, previous studies in sub-elite male kayakers have reported that, during a three-year longitudinal monitoring, improvements in all-out sprint kayak performance over 200 m and 500 m distances were observed alongside the approach and stabilization of the PU/PR at around 1.3 [4,15]. Therefore, the authors proposed it as a reference value for ameliorating paddling sprint performance (i.e., achieving a shorter time to complete the race) [4,15]. However, despite this valuable information, data regarding PU/PR in international-level medalist kayakers has not been previously analyzed. 

The main goal of this work is to describe the P–Lr between 20% and 90% one-repetition maximum (1RM) in the guided BP, guided BBP, and guided PBP in international kayakers and canoeists. The secondary aim is to compare these relationships between exercise types, and the tertiary objective is to investigate the PU/PR ratio. We hypothesize that PO on PBP is superior to pushing exercises, that there is no difference in P–Lr between BBP and BP, and that PU/PR on canoeists and kayakers is around 1.3. To achieve these goals, we applied an incremental protocol, over two complementary testing sessions, investigating PO from an absolute load of 30 kg to 100 kg in BP, from 30 kg to 95 kg in PBP, and from 20 kg to 60 kg in BBP, in a group of international medal-winning athletes. 

## 2. Materials and Methods

### 2.1. Study Design

The research is an observational pilot study design.

### 2.2. Participants

A total of 9 athletes (all males, mean ± SD; age: 21.0 ± 1.5 years; weight: 80.3 ± 4.8 kg; height: 183.3 ± 4.6 cm; kayak/canoe training experience: 8.8 ± 2.6 years; resistance training experience: 5.7 ± 1.9 years) completed the experimental sessions. All subjects were international medal-winning kayakers and canoeists specializing in sprint–endurance competitions (200 m–5000 m) who participated in the 2020 European Championship (October 2020, Poznan). The sample of kayakers consisted of national team members who secured 4 gold, 5 silver, 4 bronze medals, and multiple Final A placements (the final heat determining podium positions) at U23 World or European Championships over their careers. Their weekly training volume consisted of 2 to 3 resistance training sessions and 4 to 5 kayak training sessions conducted both on (kayak) and off-water (paddle ergometer). Each training session typically lasted between 60 and 120 min. Additionally, the values of maximum oxygen consumption, lactatemia response, and power output of eight athletes of this cohort, evaluated during a specific, off-water incremental test until exhaustion, can be appreciated elsewhere [16]. Participants were free from physical limitations, health problems, or musculoskeletal injuries that could negatively impact performance and the full range of motion in BP, BBP, and PBP. Participants were required to abstain from alcohol and exercise for 48 h before all experimental trials. After an exhaustive explanation of the aim of the research, risks, and benefits, all the volunteers have read and signed an informed consent form. The research protocol was approved by the Sport and Physical Education Ethics Committee of the University of Novi Sad, Serbia (n°47-06-02/2021-3). Data and sensible information were protected by the General Data Protecting Regulation (GDPR).

### 2.3. Procedure

#### 2.3.1. Warm-Up Protocol

Each session began with a 5 min warm-up on the kayak ergometer at an intensity between 68% and 72% of the heart rate maximum, which was previously measured during an all-out water test. Then, athletes performed 3 sets of 10 repetitions on dumbbell chest press and the exact same amount on bodyweight push-up, and 5 min of self-selected upper mobility exercises. Overall, the warm-up procedure lasted 20 min, and the incremental protocol followed in the order BP, BBP, PBP. 

#### 2.3.2. Incremental Protocol

Due to the high number of repetitions required, the study was conducted in two days, 48 hours apart, at the same time of the day. The incremental protocol comprised two complementary testing sessions, termed Test 1 (T1) and Test 2 (T2), that, when combined, allowed to obtain P–Lr. On T1, the starting absolute load was set at 30 kg for PBP and BP, while at 20 kg for BBP. On T2, it was set at 35 kg for PBP and BP and at 25 kg for BBP. Subsequently, on each testing day, the load was increased by 10 kg at every set until reaching, on T1, an absolute ending load of 90 kg for PBP, 100 kg for BP, and 60 kg for BBP, or until subjects reached their 1RM if it was below this arbitrary weight. On T2, the end of the incremental test was set at 95 kg for PBP, 95 kg for BP, and 55 kg for BBP, or again, if 1RM was reached directly. Regarding sets and repetitions, in the PBP and BP testing: five repetitions with lighter loads (≤55 kg), three repetitions with medium loads (60 kg and 65 kg), and a set consisting of a single repetition for heavier loads (≥70 kg) were used. In contrast, a single effort was monitored in the BBP due to the high repeatability of the exercise [17]. For the subsequent analysis, we considered the highest mean value for each load of T1 and T2 when more than one repetition was performed (e.g., loads ≤ 65 kg in BP and PBP). Conversely, when only a single repetition was performed (i.e., loads ≥ 70 kg for BP and PBP, and ≥20 kg for BBP), we considered the single value at each load for both T1 and T2. Regarding recovery, in BP, BBP, and PBP the inter-sets rest period was fixed at five minutes [18]. Passive rest between exercises was established at 15 min to allow a complete recovery.

#### 2.3.3. One Repetition Maximum

The direct achievement of 1RM was possible in 2 athletes regarding BP, and 4 on PBP; for the others, it was indirectly estimated through the load–velocity relationship (L–Vr) [19]. Specifically, a linear regression was fitted to the velocity V–Lr_BP_ experimentally recorded values considering a minimum velocity (V_1RM_) of 0.15 m/s to estimate 1RM in BP. In contrast, the same function, including V–Lr_PBP_ and a V_1RM_ of 0.45 m/s, was used for PBP. The 1RM for BBP press was set at 80% of the maximum weight lifted in BP [20]. 

#### 2.3.4. Calculation of the Standardized Relative Load

Once the 1RM had been directly or indirectly achieved, standardized absolute load [kg] from 20% to 90% 1RM at each 10% interval was extrapolated.

#### 2.3.5. Calculation of the Power Output at a Standardized Relative Load

**Figure 1:**
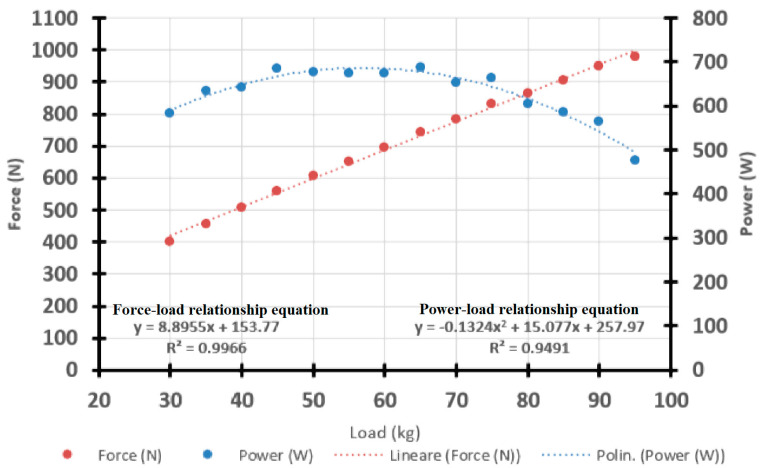
Example of data analysis and computation of the Power–Load relationship by interpolation of the experimental data.

Once the absolute load [kg] was extrapolated at the standardized percentage of 1RM, PO was indirectly calculated based on the work (*W*) performed during each movement and its execution time as PO = W/Δt where W = m × g × Δh, m represents mass in Kg, g is gravitational acceleration constant 9.8 m/s^2^, Δt is the execution time and Δh is the vertical displacement. The overall P–Lr curve was then found using the individual data and the P–Lr polynomial equation for data interpolation:

PO(m) = a*m^2^ + b*m + c

where PO indicates power in Watts. The values for the a, b, and c parameters were estimated by interpolation of the experimental data through the appropriate Microsoft Excel function. An example of such analysis for one representative subject is shown in Figure 1.

### 2.4. Exercises

Guided eccentric–concentric bench press and ballistic bench press. BP and BBP were performed on a Smith Machine (Multipower^®^, Technogym^®^, Cesena, Italy). Repetitions began with a rapid but controlled eccentric movement, immediately followed by a straight explosive vertical push. In BP, all the repetitions of the sets were performed continuously without intra-set rest. In BBP, the athlete had to let go of the barbell after each repetition.

Guided concentric-only prone bench pull. On the same Smith Machine (Multipower^®^, Technogym^®^, Cesena, Italy), PBP was built using two commercial wooden plyometric boxes of 76.2 cm (30 inch) (Lacertosus^®^, Parma, Italy) as bases, and a bench (thickness of 8 cm) was then placed on them. The bench was raised by another 20 cm to let each subject start with the arms extended during the pulling movement. Even at the lowest load, the sets were performed as a sum of single repetitions inserting a self-paced pause of approximately 2 s to eliminate the contribution of the rebound effect. 

### 2.5. Instrumentation

A linear position transducer (LPT) (MuscleLab^TM^, Model 4000e, Ergotest Technology, Bjønnveien, Norway) with a sampling frequency of 200 Hz was used for data collection, as previously carried out in similar research [3,21], to determine instantaneous displacement during the concentric phase of the lift. Mean velocity (m/s) and mean PO (W) were computed from these data. 

### 2.6. Statistical Analysis

Data were summarized by the mean and standard deviation (sd). We applied a two-way repeated-measures ANOVA to explore differences among different exercises and relative load from 20% to 90% 1RM. For ANOVAs, Mauchly’s sphericity test was performed, and where this assumption was violated, Greenhouse Geisser adjustments were used. Bonferroni correction for multiple comparisons was applied. Effect sizes (Cohen’s d) were calculated to quantify the magnitude of differences in mean PO between exercises at various %1RM. Statistical significance level was set at *p* < 0.05, analysis was performed using STATA16 (StataCorp LLC, College Station, TX, USA). Of note, due to the small sample size, the results of the statistical analyses should be considered solely for descriptive and not inferential purposes.

## 3. Results

### 3.1. Performance Characteristics of the Sample

The subjects’ average 1RM in BP, PBP and BBP was, respectively: 99.2 ± 11.1 kg, 95.1 ± 10.1 kg, and 79.3 ± 8.8 kg. Related to their average body weight (BW), 1RM was, respectively: 1.2 ± 0.1 kg^−1^, 1.2 ± 0.2 kg^−1^, and 1.0 ± 0.1 kg^−1^. Overall, PU/PR was 0.96. 

### 3.2. Power–Load Relationship

#### 3.2.1. Between Exercises

**Figure 2:**
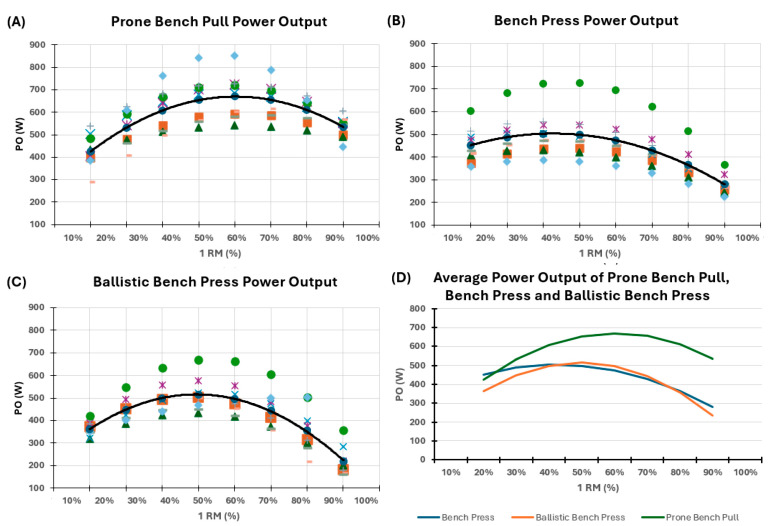
Individual values of power output for each of the three exercises, along with the average power output across the different exercises.

Overall, PBP shows the greatest values of PO compared to BP and BBP from the relative load of 40% to 90% 1RM. In contrast, amongst BP and BBP, no significant difference at any relative load was found. Post hoc analysis shows that the relative load that maximizes PO differs between exercises; 60% 1RM for PBP, 50% 1RM for BBP and 40% for BP; while the minimum PO’s relative load is 20% 1RM for PBP and 90% 1RM for BBP and PB (Figure 2, Table 1). The magnitude of differences in mean PO between exercises at various %1RM is reported in Table 2.

Figure 2 reports the individual and mean power output for the following exercises: Panel A, Prone Bench Pull; Panel B, Bench Press; Panel C, Ballistic Bench Press. Different individuals are represented with distinct symbols within the same Panel, but are constant between Panels; the black marked line of Panels A, B, and C represents the average power output. Finally, in Panel D, a graphical comparison of the mean power output across the three exercises is available. 1RM—One repetition maximum; %—Percentage; PO—power output; W—Watts. 

#### 3.2.2. Within Exercises

Within each exercise, the PO was not statistically different from 40% to 80% 1RM in PBP, from 40% to 60% 1RM in BBP, and from 20% to 60% in BP (Table 1).

## 4. Discussion

To our knowledge, this study is the first to describe P–Lr from 20% to 90% 1RM in BP, BBP, and PBP in international medal-winning canoeists and kayakers. The main finding is that P–Lr differs between PBP, BP and BBP, with the highest PO in PBP compared to BP and BBP.

### 4.1. Pull-to-Push Ratio

Our sample shows an overall PU/PR of 0.96, which, if compared with the investigation of McKean and Burkett in sub-elite kayakers (2010, 2014) could be considered low. Indeed, the cited studies found that improvements in all-out sprint kayak performance were correlated with the PU/PR ratio approaching 1.3 during three-year longitudinal monitoring. However, compared to the present study, some methodological differences about the modality of PU/PR investigation emerge. In fact, in these investigations [4,15], it was obtained by dividing the maximum weight lifted during a chin-up (plus body mass) versus 1RM in BP, while our study divided the 1RM in PBP versus 1RM in BP. Such a discrepancy may explain the different results, since, although the chin-up is a pulling exercise, the primary muscles involved act at different joint positions compared to the PBP. This hypothesis would be confirmed by observing results obtained in studies which utilized our same exercises, reporting similar PU/PR values: equal to 1.0 [22,23] or 0.92 [2]. 

Another difference could be attributed to the composition of our sample, which included kayakers specialized in sprint and endurance disciplines (i.e., kayak marathon), whereas the cited studies focused exclusively on sprint specialists. Therefore, it seems that a high PU/PR could be appropriate for sprint events, where explosive upper-body strength is critical, while in longer distances, other factors such as endurance, may play a more significant role in performance, thus reducing the overall PU/PR. However, further research is needed to determine the most effective method for calculating the PU/PR ratio through exercise and to identify discipline-specific reference values.

### 4.2. Power–Load Relationship

#### 4.2.1. Power–Load Relationship Between Exercises

The greatest values of PO in the PBP than those observed for BP and BBP, from 40% to 90% 1RM are in line with previous investigations [13,24]. This finding may reflect the sport-specific demands placed on this type of athlete. Indeed, kayakers and canoeists rely heavily on pulling motions to generate propulsion, as outlined as one of the most important characteristics of paddlers by coaches of this discipline [25]. This could contribute to greater PO development in pulling exercises like the PBP as opposed to pushing tasks such as the BP. Similarities with other studies could be explained by the fact that Sánchez-Medina et al. (2014) recruited strength-trained athletes mainly from combat sports (judo and wrestling) [13], and Pearson et al. (2009) a group of sailors [24], both activities that require a strong pull, the first to immobilize the opponent, while the second to pulling ropes to control the sails. Moreover, the difference in the PO between pushing and pulling exercises might also derive from different mechanical advantages during execution; indeed, we know that BP, unlike PBP, has a sticking point that could be up to half of the concentric effort [13]. Finally, no statistically relevant difference was found between BP and BBP in the P–Lr as assumed.

#### 4.2.2. Power–Load Relationship Within Exercises

In PBP, the maximum PO was found at 60% 1RM, with non-significantly different values between 40 and 80% 1RM. This result slightly differs from previous research that observed the maximum at 78.6 ± 5.7% 1RM without distinction between 60 and 100% 1RM [24] or at 70 ± 4% without difference in the range 50–90% 1RM [13]. This difference may be attributed to the fact that, the strength-trained athletes in Sánchez-Medina et al. (2014) may have been able to optimize their annual training specifically to enhance PO and strength in pulling exercises [13]. In contrast, international-level kayakers typically engage in twice as many on- and off-water sessions compared to resistance training sessions, potentially limiting the extent of PO adaptations due to reduced RT volume. In contrast, the entire P–Lr is consistent with that measured in elite rowers [21]. Maximum PO is comparable to those of a Spanish Olympic rowing championship team [21]; however, from the overall P–Lr, we assume that the Spanish team trains more with a heavy relative load than our sample because their maximum PO was attained near 75% 1RM. Indeed, training with a high percentage of 1RM shifts the bell-shape P–Lr to the right [26]. This difference could be partially explained by the relatively lower competitive level of our sample. Although all participants were international-level medalists, none had competed in the Olympic Games. Consequently, they lacked access to the training volume, recovery strategies, and support systems typically available to Olympic-level athletes, who may have modestly limited their overall physical conditioning and adaptations.

In BP, the highest PO occurred at 40% 1RM and was not significantly different from the values reported between 20% and 60% of 1RM. This result slightly differs from previously published data in a cohort of strength-trained athletes, primarily from combat sports such as judo and wrestling, that found the maximum PO at 56 ± 2%, without differences from 40% to 70% 1RM [13]. We hypothesize that the athletes in our research use lighter loads on the bench press than the cited sample because the pushing exercise is not the primary goal in paddle sports. Indeed, training with a lighter relative load shifts the P–Lr to the left with the expected highest PO to move toward the lower %s of the curve [26]. Moreover, it can be speculated that the specific loads encountered during sport participation influence PO adaptation. Indeed, combat athletes require higher pushing forces to quickly overcome an opponent’s resistance and avoid being controlled or taken down, demanding powerful pushing actions and as suggested before, they may have been able to optimize their annual training specifically to enhance PO and strength in this movement [13]. Of note, the pushing action in paddling does not have the same fundamental importance for specific on-water performance. In our investigation, the maximum PO in BBP peaked at 50% 1RM, without a significant difference from 20% to 60% 1RM. Our result is similar to the one performed on professional rugby players that have found a non-statistical difference in PO from 20% to 60% 1RM, even if it was shown that their maximum PO was at 30% 1RM [14]. The difference in maximum PO could be due to the different sports as well as training regimes. In this context, when comparing two cohorts from different sports that prioritize sport-specific (on-pitch and on-water) training over RT in terms of volume, our results are consistent—and in this case superior—in terms of the percentage in which maximum PO is achieved. This further reflects the high performance level of our participants.

##### Possible Elements for Power-Based Resistance Training Prescription

Considering that during the paddling stroke the paddle blade is submerged in water and acts as a fixed point of resistance, the muscle action involved in pulling closely resembles a contraction against a high resistance. Therefore, contextualizing the result in our sample of kayakers, it would be ideal to obtain maximum PO at higher relative loads (such as the one reported in a rowing championship team [21]), because this indicates the ability to generate greater PO under conditions that closely mimic the high-resistance demands encountered during actual paddling. This ability reflects a superior capacity to apply strength and power effectively during the phases of the stroke, which are critical for maximizing propulsion and overall performance. To achieve this, training should focus on loads that shift the maximum PO of the PBP toward higher intensities [26]. Therefore, from a strength training perspective, this would likely be accomplished by training within the higher portion of the P–Lr (i.e., 60–80% 1RM) and even near-maximal loads (≥85% 1RM). Additionally, we suggest focusing on the higher end of the P–Lr, as training at these loads is more likely to enhance strength and neuromuscular adaptations [27].

Moreover, since we have observed that PO is maximized at 60% 1RM (669.66 ± 98.2 W) in PBP, and that paddlers generate 747 ± 151 W during an all-out start of 10 s [12], prescribing training loads around 50–70% of 1RM in PBP may be effective for improving this part of the race. Specifically, coaches must count the number of strokes performed at maximal intensity during the initial start, which could be approximately 20. With that information in mind, coaches could prescribe to athletes several long sets performing the exact number of pulling movements (i.e., 3 sets per 20 repetitions, with 5 min of rest in between) or could opt to break down these long sets into smaller groups of reps with short rest periods in between (10 to 30 s) [28] and, again, complete rest between sets. For example, a set of 20 repetitions can be broken into a set of four blocks of five repetitions with 20 s of rest between blocks (i.e., 3 sets × (4 blocks × 5 reps, 20 s rec)/5 min). This approach would maintain PO mainly constant throughout all the repetitions of a set to increase muscle adaptations and minimize neuromuscular fatigue [28]. 

Moreover, considering that a PO of 424.64 ± 74.93 W was observed at 20% 1RM in PBP, and that paddlers generate 374 ± 63 W in the first half and 323 ± 36 W in the second half of a 2 min all-out kayaking performance [12], a training prescription aimed at simulating the full race effort could be developed based on these values. For example, during the competitive phase, coaches might prescribe a full, 2 min set on the PBP using lighter loads (i.e., 20–30% 1RM) to target in-water power–endurance. However, during the preparatory phase, this could be structured as repeated sets of 30 s of pulling movements followed by 30 s of rest, continued until a total of 2 min of RT is completed. 

Another approach to closely simulate race demands involves starting with a load corresponding to the all-out start PO (60% 1RM), and then progressively reducing the load as PO declines, allowing the athlete to complete the 2 min set. In this scenario, teammates assist by deloading the barbell for the athlete performing the exercise, minimizing dead time between repetitions. 

Regarding exercise selection, considering that paddling is a predominantly pulling activity, from our perspective, PBP must have priority in all the RT sessions. However, BP and BBP must be implemented to avoid upper-body’s agonist and antagonist muscle imbalances.

##### Limitations and Future Directions

The major limitations of this study are the number of athletes recruited (*n* = 9) and the measurement of a guided exercise. The restricted cohort of international-level medal-winning athletes has been analyzed for descriptive purposes only and does not allow for drawing broader inferences to a cohort of matched kayakers, nor comparisons with paddlers of different levels (sub-elite, Olympic paddlers). Moreover, no gender stratification was possible. For these aims, future studies on larger cohorts, better representing both sexes, and involving groups of paddlers of different levels would be necessary. For the measurement of a guided exercise, we chose this modality because the BBP is usually performed on a guided machine due to the potential danger associated with its execution with a free barbell.

Moreover, future investigations on PU/PR should consider standardizing the methodology (i.e., exercises from which this ratio is calculated), along with the identification of discipline-specific (i.e., kayak sprint versus kayak endurance competitions) reference values. Additionally, the exercise order was not randomized because all athletes were tested simultaneously (i.e., in the same training session) due to time constraints imposed by a rigid calendar of structured training. Additionally, the exercise order was chosen because, for the PBP, we needed to completely change the starting position of the barbell and set up the bench using two wooden plyometric boxes, as described in the method. Therefore, it would have been impossible to quickly and repeatedly change this setup after a bench press set by another athlete. Therefore, a future study that will randomize the exercise order would add scientific rigor.

Finally, a dedicated study that answers how a training prescription at peak PO might impact sprint kayak performance in international-level elite kayakers is warranted, along with one aiming to correlate gym-based P–Lr profiles with on-water paddling stroke characteristics (force, velocity, and power). Such investigations would enhance understanding of how RT adaptations transfer to paddling performance, thereby refining training specificity for sprint kayak athletes. 

## 5. Conclusions

This study is the first to describe the P–Lr from 20% to 90% 1RM in BP, BBP, and PBP in international-level, medalist-winning canoeists and kayakers. The main findings are that pulling in PBP showed an overall higher PO than the pushing movement (BP and BBP), and that the maximum PO was found at 60% 1RM in PBP, 40% 1RM in BP, and 50% 1RM BBP. Moreover, our elite cohort of paddlers has a PU/PR < 1. Although necessitating confirmation in a wider sample, these data may provide preliminary reference values useful to prescribe athlete-specific power-based oriented training in paddle-sports. 

## Figures and Tables

**Table 1:** Values of average power output across different loads (from 20% to 90% 1RM) and exercises.

Different Loads	20%	30%	40%	50%	60%	70%	80%	90%
	a	b	c	d	e	f	g	h
Prone bench pull-1, (*n* = 9)								
mean ± sd	424.64 ± 74.93	531.41 ± 77.24	607.83 ± 93.53	653.92 ± 102.56	669.66 ± 98.2	655.05 ± 79.73	610.11 ± 51.20	534.82 ± 47.72
Loads comparison	a vs. all	b vs. a,d,e,f	c vs. a	d vs. a,b,h	e vs. a,b,h	f vs. a,b,h	g vs. a,h	h vs. a,d,e,f,g
Bench press-2, (*n* = 9)								
mean ± sd	452.10 ± 76.81	487.64 ± 90.36	503.11 ± 98.80	498.51 ± 100.99	473.84 ± 96.49	429.10 ± 85.14	364.29 ± 66.99	279.41 ± 42.72
Loads comparison	a vs. g,h	b vs. f,g,h	c vs. f,g,h	d vs. f,g,h	e vs. g,h	f vs. b,c,d,g,h	g vs. f,h	h vs. all
Ballistic bench press-3, (*n* = 9)								
mean ± sd	363.48 ± 34.79	448.08 ± 50.74	498.35 ± 63.89	514.29 ± 71.18	495.91 ± 75.23	443.19 ± 82.40	356.14 ± 100.90	234.76 ± 135.48
Loads comparison	a vs. b,c,d,e,f,h	b vs. a,d,g,h	c vs. g,h	d vs. a,f,g,h	e vs. a,g,h	f vs. a,d,g,h	g vs. b,c,d,e,f,h	h vs. all
Exercises comparison	1 vs. 3	1 vs. 3	1 vs. 2, 3	1 vs. 2, 3	1 vs. 2, 3	1 vs. 2, 3	1 vs. 2, 3	1 vs. 2, 3

**Table 2:** Cohen’s d Effect Sizes for mean power output across different %1RM in prone bench pull, bench press, and ballistic bench press.

Exercise	Percentage of One Repetition Maximum							
	20%	30%	40%	50%	60%	70%	80%	90%
PBP vs. BP	−0.36	0.52	1.08	1.52	2.01	2.73	4.12	5.64
PBP vs. BBP	1.05	1.27	1.36	1.57	1.98	2.62	3.22	5.56
BP vs. BBP	1.50	0.53	0.05	−0.17	−0.25	−0.16	0.09	1.09

PBP—prone bench pull; BP—bench press; BBP—ballistic bench press.

## Data Availability

The data associated with the paper are not publicly available but are available from the corresponding author on reasonable request.
